# Therapeutic Implication of miRNAs as an Active Regulatory Player in the Management of Pain: A Review

**DOI:** 10.3390/genes15081003

**Published:** 2024-07-31

**Authors:** Mubashir Hassan, Saba Shahzadi, Muhammad Yasir, Wanjoo Chun, Andrzej Kloczkowski

**Affiliations:** 1 The Steve and Cindy Rasmussen Institute for Genomic Medicine at Nationwide Children’s Hospital, Columbus, OH 43205, USA; saba.shahzadi@nationwidechildrens.org (S.S.); andrzej.kloczkowski@nationwidechildrens.org (A.K.); 2 Department of Pharmacology, College of Medicine, Kangwon National University, Chuncheon 24341, Republic of Korea; yasir.khokhar1999@gmail.com (M.Y.); wchun@kangwon.ac.kr (W.C.); 3 Department of Pediatrics, The Ohio State University School of Medicine, Columbus, OH 43205, USA; 4 Department of Biomedical Informatics, The Ohio State University, Columbus, OH 43210, USA

**Keywords:** miRNAs, pain, receptor, pain management

## Abstract

Chronic pain is frequently associated with neuropathy, inflammation, or the malfunctioning of nerves. Chronic pain is associated with a significant burden of morbidity due to opioid use, associated with addiction and tolerance, and disability. MicroRNAs (miRs) are emerging therapeutic targets to treat chronic pain through the regulation of genes associated with inflammation, neuronal excitability, survival, or de-differentiation. In this review, we discuss the possible involvement of miRs in pain-related molecular pathways. miRs are known to regulate high-conviction pain genes, supporting their potential as therapeutic targets.

## 1. Introduction

Pain receptors, also known as nociceptors, are specialized nerves that detect and transmit signals in response to potentially harmful stimuli such as temperature extremes, tissue injury, pressure, or irritants [1,2,3]. Upon the activation of pain receptors by a noxious stimulus, they transmit action potentials to spinal interneurons and, in turn, to the brain, where the signal may be interpreted as pain [4,5]. This initiates aversive responses such as pulling away from a hot surface or conscious perceptions of pain [6]. Different subtypes of nociceptors respond preferentially to mechanical, thermal, or chemical stimuli [7]. Furthermore, activation of nociceptors triggers inflammatory responses that both sensitize nociceptors and contribute to the perception of pain.

Generally, nociceptors express membrane receptors that detect heat, cold, or different types of hazardous substances [8]. Activation of these receptors promotes nociceptive action potentials, characteristically in unmyelinated C-fibers and myelinated Aδ-fibers [9,10], and releases excitatory neurotransmitters onto interneurons in the spine (especially the dorsal horn) [9,10]. The spinal dorsal horn’s excitatory and inhibitory interneurons can influence the perception of pain associated with nociceptive active potentials [11,12]. These physiological pain responses protect against tissue damage by triggering aversive behaviors. However, increased activity and responses of spinal dorsal horn neurons—a phenomenon known as central sensitization—are frequently linked to persistent pathological pain, including neuropathic and inflammatory pain [13,14]. Central sensitization forms the cellular basis for hyperalgesia (extreme sensitivity to pain) and allodynia (pain due to a stimulus that does not normally provoke pain) [15,16]. Alternatively, chronic peripheral activation of nociceptors can also lead to chronic pain, a condition that can be difficult to treat and can have a significant impact on quality of life.

MicroRNAs (miRs) are non-coding, tiny RNA molecules that control post-transcriptional regulation of gene expression. The biosynthesis of miRs is divided into two main steps: transcription and processing [17,18]. The first step in miR biosynthesis is the transcription of the DNA sequence encoding the miR by RNA polymerase II to produce a primary miR (pri-miR) transcript. Pri-miRNAs are typically several hundred nucleotides long and contain a stem-loop structure, with one end of the stem-loop being a single-stranded RNA tail [19]. The pri-miR transcript is processed by two RNase III enzymes, Drosha and Dicer, to generate the mature miRNA. First, the pri-miRNA is cleaved by Drosha in the nucleus to produce a precursor miR (pre-miR), which is typically 70–100 nucleotides long and contains a hairpin structure. After being transferred to the cytoplasm, the pre-miR undergoes further processing by Dicer to create a double-stranded RNA duplex. This duplex’s other strand, referred to as the passenger strand, is often eliminated while the other strand becomes the mature miR and is integrated into the RISC [20].

Pain management often utilizes medication to alleviate or control pain [21]. Nonsteroidal anti-inflammatory drugs (NSAIDs), such as aspirin, ibuprofen, and naproxen, are usually used as first-line therapy [22,23]. NSAIDs are associated with gastrointestinal issues, stomach irritation, ulcers, and gastrointestinal bleeding [24]. Furthermore, prolonged use of NSAIDs may increase the risk of heart attacks and strokes [25]. Finally, NSAIDs can also lead to kidney problems, particularly in individuals with pre-existing kidney disease [26]. These adverse effects both contribute to population-wide morbidity due to the wide use of NSAIDs and limit the use of NSAIDs in multimorbid patients [27].

Opioids, including oxycodone, hydrocodone, and morphine, are also sometimes used [28]. However, these drugs have a high potential for abuse, addiction, and physical dependence. Moreover, high doses or misuse can lead to slowed breathing and respiratory depression. Opioids also cause constipation and may cause drowsiness and impair cognitive function [29,30]. Finally, while not the sole cause, prescription opioids do contribute to the opioid overdose crisis [31]. Acetaminophen is an effective mild analgesic and antipyretic agent frequently used for pain management [32]. Reports shows that acetaminophen exhibited serious drawbacks, including liver damage, and is less effective in reducing inflammation compared to NSAIDs [33].

Neurotransmitter modulating agents, including amitriptyline and duloxetine, are also used in pain management. In neuropathic pain, anticonvulsants, including especially gabapentin and pregabalin, are typically first-line therapies [34]. For both these classes of drugs, side effects can include dizziness, drowsiness and weight gain [35]. In spite of these numerous therapies, the numerous risks and limited efficacy leave a tremendous unmet need for novel analgesic therapies [36].

## 2. Role of miRs in Pain

miRs are involved in the post-transcriptional regulation of genes [37] by binding to complementary sequences in target mRNAs. miR binding either inhibits translation or promotes mRNA degradation. Recent studies have shown that miRs modulate pain perception by regulating the expression of target genes involved in pain signaling pathways, including in nociceptors and spinal interneurons [38].

### 2.1. miR-124a

miR-124a is a key player in the spinal cord [39]. In a subset of spinal neurons in animal models, intraplantar formalin injection causes a considerable drop in miR-124a levels [40,41]. Moreover, miR-124 injection in the spinal cord decreases second-stage formalin-induced behaviors [38]. An independent study has shown that miR-124 directly regulates the expression of methyl-CpG binding protein 2 (MeCP2) [42]. MeCP2 is highly expressed in mature nerve cells and works as a transcriptional regulator of additional inflammatory pain genes [43,44].

Furthermore, it has been shown that the brain-derived neurotrophic factor (BDNF) protein, which is also well known as an established target gene of MECP2 that promotes nociceptive transmission in the dorsal horn, is downregulated by miR-124. Consequently, neuropathic pain following spinal cord damage may be influenced by miR-124 downregulation. Conversely, it has been demonstrated that miR-124 enhances microglia quiescence [45].

miR-124a has been shown in another investigation to suppress the expression of transient receptor potential vanilloid 1 (TRPV1), a crucial receptor involved in pain perception [46]. TRPV1 is a neuronal cation channel associated with sensitization to noxious stimuli, contributing to pain and inflammation [47]. Another mouse study found that glial released inflammatory factors during the central sensitization phase of neuropathic pain were linked to the shift in TRPV1 expression in adult mice’s cortical neurons. In this study, neuropathic pain was constitutively suppressed during early life by anti-inflammatory neuroimmune regulation [48]. In a second study, blood samples from patients with diverse neuropathic pain syndromes, such as polyneuropathy, postherpetic neuralgia, and trigeminal neuralgia, were found to express certain miRs, such as miR-124a and -155, differently in comparison to healthy persons [49]. Histone deacetylase sirtuin1 (SIRT1), a negative regulator of Foxp3, a master regulator of the formation of Tregs (regulatory T cells), is directly repressed by miR-124a and -155. Increased levels of miR-124a and miR-155 promote the differentiation of Tregs, which have an anti-inflammatory role, in neuropathic pain [50]. Additionally, the cerebral fluid of cancer patients who experienced pain showed downregulation of miR-124, indicating that miR-124 may be a useful analgesic medication for treating cancer pain sufferers [51].

### 2.2. miR-103

miR-103 is the first miR characterized in neuropathic pain [49]. In these reports, miR-103 functionally modulates nociceptors via transcripts, including calcium transients and their subunits (Cav1.2-α1, -α2δ1 and -β1) [52,53]. In neuropathic pain models, spinal dorsal horn neurons exhibit downregulation of miR-103, indicating that miR-103 may be a potential target for pain [54]. miR-103 has also been implicated in the regulation of the mu-opioid receptors (MORs). Inhibition of miR-103 led to increased MOR expression and enhanced the analgesic effects of opioids [55,56].

### 2.3. hsa-mir-548

The human miR-548 family contains 69 members located in almost all chromosomes [57]. A functional enrichment analysis of miR-548 gene family targets implicates a range of biological processes and diseases [58]. It has been reported that hsa-miR-548 expression is linked with the impaired interferon (IFN) signaling pathway in chronic hepatitis B (CHB) [59,60]. During the immunological activation (IA) phase of CHB, expression of hsa-miR-548-5p increases [59]. miR-548 may downregulate host antiviral response via direct targeting of IFN-λ1 [61], but miR-548 has been implicated in Wnt, MAPK, and TGF-β pathways as well [62]. According to another study, the 3′UTR of IFN-λ1 is the target of the miR-548 family members, which include miR-548b-5p, miR-548c-5p, miR-548i, miR-548j, and miR-548n. MiR-548 mimics inhibited IFN-λ1 expression, and, by controlling the host’s antiviral response, miR-548 could potentially be a treatment option for viruses [63].

### 2.4. miR-143

miR-143 has been implicated in the development and function of pain-sensing neurons in mice [64]. miR-143 is significantly downregulated (13.7 times) in tumors compared to the matched control that examined the function of miRs in pain and inflammation. [65]. Abnormal expression in miR-143 has been found in rheumatoid arthritis (RA) patients [66,67]. Fibromyalgia patients had decreased expression of hsa-miR-143-3p [68]; miR-143 also degrades COX-2 mRNA and regulates pain mediator production in pancreatic cancer [69]. After receiving a full Freund’s adjuvant (CFA) injection, ipsilateral Dorsal Root Ganglions (DRGs) showed noticeably reduced miR-143 expression levels [64]. Moreover, a bioinformatics analysis shows that around 1305 genes are associated with miR-143, most importantly mitogen-activated protein kinases MAPK7, MAPK3, COX2, matrix metalloproteinase, and TNF, all prominent target genes. This shows the significance of miR-143 in pain because all these target proteins are directly linked with various pain signaling pathways and the expression of miR-143 may control the expression of these target proteins [70].

### 2.5. miR-146a

miR-146a has been found upregulated in response to inflammatory stimuli and implicated in the negative feedback regulation of the inflammatory response [71,72,73]. miR-146a inhibits the production of cytokines and pain perception by targeting the mRNA of multiple pro-inflammatory genes, such as interleukin 1 receptor-associated kinase 1 (IRAK1) and tumor necrosis factor receptor-associated factor 6 (TRAF6) [74,75]. It has been proposed that the expression of Toll-like receptors (TLR) and nuclear factor kappa B (NF-κB) influences osteoarthritis pain [76]. These inflammatory response genes are regulated through the binding of mir-146 to the 3′-UTR (the three prime untranslated regions), inhibiting protein translation and reducing mRNA stability [77]. Therefore, miR-146 may act to reduce osteoarthritis pain driven by inflammatory cytokines [77,78]. In other pain models, a miR-146a-5p mimic inhibits TRAF6/c-Jun N-terminal kinases (JNK)/chemokine (C-C motif) ligand 2 (CCL2) signaling in astrocytes. miR-146a-5p inhibits spinal nerve ligation (SNL)-induced mechanical allodynia and spinal TRAF6 expression [79].

### 2.6. miR-let-7b

miR-let-7b is mainly involved in the transmission of pain signals in human spinal cord neurons [80]. In another study, the functional role of extracellular miRs in interaction with various receptors such as Toll-like receptor 7 (TLR7) and Transient receptor potential ankyrin 1 (TRPA1) to elicit pain, has been explored [81]. In DRG neurons, miR-let-7b causes fast inward currents and action potentials [80]. The most significant effect of let-7b is that it causes TLR7/TRPA1-dependent single-channel activity in HEK293 cells that overexpress TLR7/TRPA1 [82,83]. Let-7b injected in an intraplantar manner causes quick, spontaneous pain through the TLR7 and TRPA1 receptors. Ultimately, neuronal stimulation of DRG neurons can release let-7b and let-7b inhibitor diminishes formalin-induced TRPA1 currents and spontaneous pain [84]. Furthermore, it has been discovered that miR-let-7d controls the expression of the chemokine receptors CCR2 and CX3CR1, respectively, which are connected to pain and inflammation [85].

### 2.7. miR-21

miR-21 is linked with the activation of immune cells and regulation of both neuropathic pain and barrier disruption [86]. In peripheral neuropathy models, miR-21-5p is upregulated in DRG neurons [87]. It has also been observed that miR-21 levels increase in painful polyneuropathy both systemically in white blood cells and locally in sural-nerve biopsies [88]. miR-21-5p is believed to target a range of essential pain genes, including Matrix metalloproteinases (MMPs), phosphatase and tensin homolog (PTEN), and reversion-inducing cysteine-rich protein with Kazal motifs (RECKs) [89]. MMPs like MMP9 have a role in both barrier breakdown and the start of neuropathic pain [90]. Moreover, miR-21 may positively regulate transforming growth factor beta (TGF-β), an anti-inflammatory cytokine involved in barrier impairment and implicated in neuropathic pain [91,92,93]. TGF-β1 has been proposed as a critical mediator of nociception by inhibiting the neuroimmune feedback of both neurons and glia and by promoting the expression of endogenous opioids within the spinal cord [94].

### 2.8. miR-30c

miR-30c has been implicated in the neuropathic pain in mice [95]. Several pseudo receptors were found to be lacking in mice models with antiallodynic effects, including bone morphogenetic protein (BMP), activin membrane-bound inhibitor (BAMBI), and transforming growth factor-β (TGF-β) [96]. It has been observed that miR-30c is upregulated in the spinal cord, DRG, cerebrospinal fluid (CSF), and plasma, and miR-30c-5p expression positively correlates with allodynia [97]. In patients suffering from leg-ischemia-driven neuropathic pain, miR-30c-5p expression was increased in plasma and CSF compared to control patients without pain [97].

The miRs directly linked with different targets through differential expression in various tissues have been listed in Table 1.

## 3. miRs and Pain Signaling Pathways

**Figure 1:**
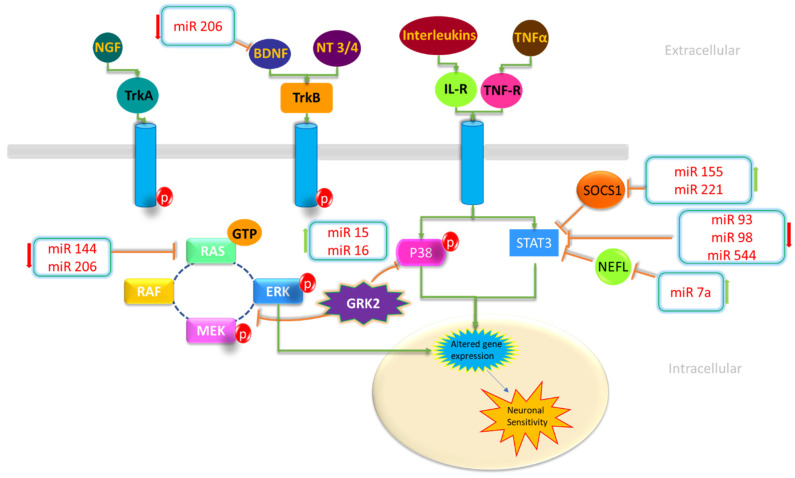
Role of miRs in altered gene expression and pain receptors [49]. Diagram showing the targets of miRs that are dysregulated in neuropathic pain and how they are implicated in intracellular signaling. The miRs (miR144, miR206, miR93, miR98, miR544, and miR206) are downregulated during neuropathic pain, according to red downward arrows; in contrast, upward green arrows indicate the elevation of miRs (miR15, miR16, miR155, miR221, and miR7a) during neuropathic pain.

Pain is primarily caused by four fundamental mechanistic processes: transduction, transmission, modulation, and perception [115]. Transduction is a process in which tissue-damaging stimuli activate nerve endings [116], and transmission is a transfer of a signal from the site of tissue injury to the brain regions that enable perception [117]. A brain function known as modulation works to selectively lower transmission system activity [118]. Perception is the subjective understanding governed by sensory signals [119]. Pain signaling pathways include ion channels, receptors, and intracellular signaling molecules that govern pain sensation with respect to causative factors. Certain miRs can regulate the chain reaction of signaling pathways. For instance, it has been demonstrated that miR-124 controls the expression of the N-methyl-D-aspartate (NMDA) receptor, which is essential for the emergence of chronic pain. The NMDA receptor, which is found in the spinal cord, is a therapeutic target for the treatment of neuropathic pain and plays a crucial role in nociceptive transmission and synaptic plasticity [120]. In some cases of neuropathic pain, it has been demonstrated that increased NMDA receptor activity leads to central sensitization. Thus, in animal models of neuropathic pain brought on by nerve damage, NMDA receptor antagonists can lessen hyperalgesia and allodynia [120]. Additionally, both miR-128a and miR-146a have also been involved in the regulation of voltage-gated sodium channel (Nav1.7) expression and intricate in pain signaling [121]. miRs can also regulate the expression of cytokines and chemokines that are involved in inflammation and pain. For instance, miR-146a has been shown to negatively regulate the expression of interleukin-1 receptor-associated kinase 1 (IRAK1), which plays a central role in the production of pro-inflammatory cytokines [122]. The overall scheme of this mechanism is shown in Figure 1.

### 3.1. miRs and Pain Mechanism

Many molecular mechanisms of pain have been associated with miRs, including brain plasticity and neurogenesis, nociceptor excitability, and chronic pain disorders [123,124]. Since brain-derived neurotrophic factor protein (BDNF) has been shown to modulate nociception, it is plausible that upregulating miR-132 in response to BDNF could enhance dendritic morphogenesis and arborization in the nociceptive pathways, thereby increasing the transmission of pain signals [82,125]. In another mouse study, it was observed that miR-29a/b plays a significant role in dendritic spine remodeling and synaptic plasticity [126]. Adult mice were given psychoactive substances like nicotine or cocaine in this experiment, and the outcomes demonstrated that miR-29a/b was consistently overexpressed in different brain areas. Furthermore, miR-29a/b was found to target the protein Arpc3, which is involved in actin branching during dendritic spine formation [127,128].

The therapeutic role of miRs in chronic pain has been revealed in earlier studies [40,129,130]. Numerous miRs, including miR-10a, miR-29a, miR-98, miR-99a, miR-124a, miR-134, and miR-183, have been significantly downregulated in the neurons of the ipsilateral trigeminal ganglion in an inflammatory rat model of CFA-induced muscular pain [124,131]. It was concluded that this downregulation of miRs increased the expression of multiple “pain-related” proteins, facilitating the onset of inflammation and allodynia [124]. On the other hand, mice with carrageenan-induced facial inflammation are shown to have an upregulated pair of miRNAs (miRNA-155/-223) in the pre-frontal cortex. Additionally, in this inflammatory model, an inverse association has been identified between miR-155 and the transcription factor, CCAAT/enhancer binding protein β [82]. In another mouse peritonitis model with acute inflammation, the enhanced levels of miR-21, miR-146b, miR-208a, miR-203, miR-142, miR-302d, and miR-219 were counter regulated by using resolvin D1 (RvD1; C22H32O5), a lipid mediator derived from docosahexaenoic acid that possesses anti-inflammatory and neuroprotective properties [132]. This study clearly shows that specific miRs act as post-transcriptional regulators of the inflammatory pain condition [133].

### 3.2. miRs in Visceral Pain

**Figure 2:**
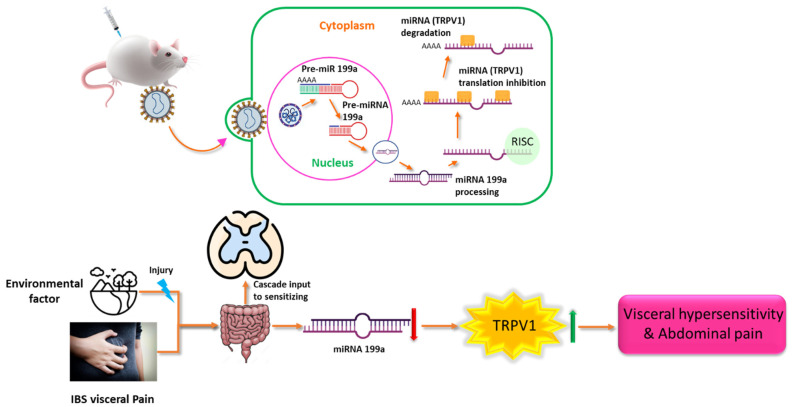
miR-199, TRPV1, visceral hypersensitivity, and visceral pain may have a mechanistic link. Several potentially special benefits over traditional retroviral gene delivery methods are provided by lentiviruses, which deliver miRs in vivo. The primary functions of these are to infect non-dividing cells, including dorsal root ganglion neuron cells, and to produce stable, long-lasting gene/miRNA expression. This work delivered and overexpressed miR-199a to suppress the expression of TRPV1 using lentiviruses. Pictures depict the associated molecular pathways. Lower left panel: Stressors in the environment cause damage to the stomach, which starts a chain reaction that makes the body hypersensitive to certain stimulants. A reduction in miR-199 and an increase in TRPV1 expression could be indicative of this hypersensitive condition [99].

Visceral pain is the condition in which internal organs become inflamed, diseased, damaged, or injured, such as bladder pain, endometriosis, irritable bowel syndrome, and prostate pain [134,135]. Published data indicates that greater expression of miR-29a in colon tissues and blood micro-vesicles of persons with irritable bowel syndrome (IBS) is positively connected with improved intestinal membrane permeability [136]. It is well known that miR-29a inhibits the production of glutamate ammonia ligase (GLUL) by interacting with complementary binding sites at the 3′UTR. This increases intestinal permeability and eventually results in persistent visceral pain [136]. Increased expression levels of miR-328, miR-320, miR-449b, and miR-500 have been linked to the downregulation of neurokinin-1 (NK1) receptors in bladder biopsies in a study of individuals with bladder pain syndrome (BPS) [109]. Other published data indicate the possible relation of miR-199a-5p with urothelial permeability in bladder pain syndrome [137]. Another possible role of miRs has been observed in patients with painful endometriosis [138,139]. Additional studies have shown that miR-181, miR-216, and miR-203 are the known targets in regard to the GABAAα-1 gene by using a luciferase reporter assay [113,140]. The overall mechanism is depicted in Figure 2.

### 3.3. Polymorphisms of miRs and Pain

**Figure 3:**
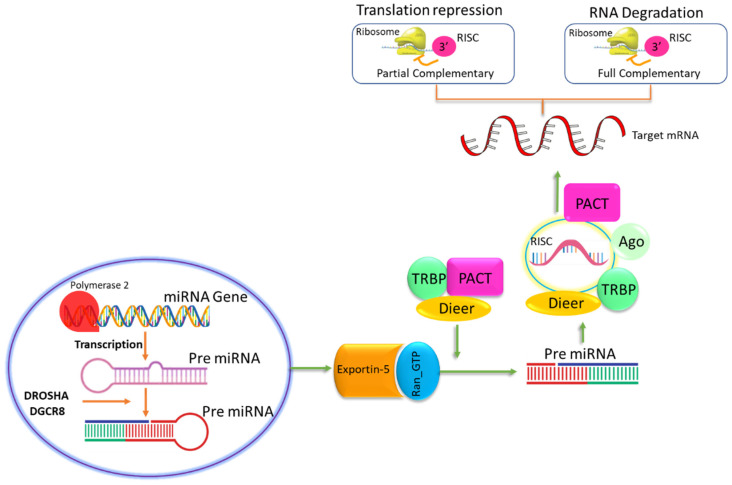
Polymorphism effect of miRs. The figure shows the formation of miRs and their polymorphism effects, like translation repression and DNA degradation, by targeting mRNA.

The change in the miRNA expression leads to genetic mutations which are associated with the function of particular genes [141]. However, there are still many studies that explore the significant impact of polymorphisms in miR genes and/or their targets, which are closely related to nociception. The single nucleotide polymorphisms (SNPs) in miRNAs are associated with the pathogenesis of different diseases. Furthermore, SNPs may also affect the functions of genes through the alteration in miRNA expression. Analogously, SNPs in the miRs’ target sites have the ability to modify or disrupt the miRs’ already-existing binding sites as well as to generate new ones [142]. It has been noted that rheumatoid arthritis and miR polymorphism are directly related. The significant correlations between psoriatic arthritis (PsA) and miR-146a with rs2910164 (G/C) have been observed in both heterozygous and dominant models. Additionally, the heterozygous model demonstrated a strong correlation between ankylosing spondylitis (AS) and the miR-146a rs2910164 (G/C) polymorphism [143]. The overall depiction is shown in Figure 3.

## 4. miRNAs as Therapeutic Targets

Multiple direct and indirect approaches have been utilized to target miRs expression in pain [144,145]. The direct strategies directly use the oligonucleotides or virus-based constructs to either block the overexpression of miRNAs or to substitute for the loss of expression of miRNAs [146]. The indirect strategies are linked with the utilization of drugs to modulate miRNA expression by regulating their transcription and processing [147]. Nucleotide sequences mimic mature or pre-miRNAs to overexpress miRNAs, while anti-sense oligos (anti-miRNAs), miRNA sponges, or miRNA decoys are typically used to limit miRNA expression [82]. The most important task is to deliver miRNAs into the central nervous system (CNS), which is the blood–brain barrier (BBB). However, there are a few techniques for CNS gene silencing, including germline transfection, lentiviral vector-mediated administration, and stereotaxic administration [148,149]. An alternative method is based on immunoliposomes, which are a combination of liposomes, tiny hairpin RNA expression plasmids, and a monoclonal antibody directed toward a specific receptor. Immunoliposome-based targeted delivery makes it easier for conjugated RNA to pass across the blood–brain barrier and enter particular brain areas [150,151].

### miRs as Prognostic Biomarkers of Pain

Biomarkers are measurable characteristics of a disease state and are crucial components for more precisely delivering tailored therapy. Furthermore, dysregulated miR expression in bodily fluids is being investigated as a non-invasive clinical diagnostic for pain [152,153,154,155]. Multiple studies have explored changes in miRNA expression in the inflammatory pain-affected tissue samples in rodent models [88,155,156]. It has also been observed that miRs such as miR-103, miR-124a, miR-203, and miR-7a have a direct influence on the management of pain [52,157].

The association among pain, plasma cytokines, and miRs in blood has been detected [158]. In this study, miRs expression level investigations were used to examine twenty-two controls, thirty-three individuals with persistent pelvic discomfort, and twenty-three individuals with Vertebrobasilar dolichoectasia (VBD), a disorder marked by elongation, tortuosity, and ectasia of the basilar artery [158]. VBS may manifest clinically by compression of the cranial nerves, ischemic symptoms, or intracranial bleeding and irritable bowel syndrome (IBS). It has been noted that miR dysregulation in VBD is expected to impact estrogen-relevant pathways, which may play a role in pelvic-localized pain. Moreover, changes in muscle, neuron, and glial cell function have been linked to miRNA abnormalities in both VBD and IBS patients, which in turn affect pain [158]. miR-132 modulates cholinergic signaling and inflammation in human inflammatory bowel disease. Compared to healthy controls, IBD patients had higher levels of miR-132 and, accordingly, decreased acetylcholinesterase (AChE) activity. These findings support the idea that inflammation plays a homeostatic function in cholinergic transmission [159,160].

## 5. miRs-Gene Association Networks in Pain

Numerous miRs have been found to be connected to genes involved in chronic pain. Dysregulation of these miRs can affect the expression of genes related to neuroinflammation, neural plasticity, and pain signaling. Table 2 lists some known instances of miRs associated with genes linked to chronic pain.

## 6. Conclusions

Our review highlighted basic mechanisms of miR regulation of pain through a range of molecular targets expressed primarily in neurons. The expression levels of miRs are characteristic of neuronal encoding of different pain states. miRs play an essential role in the molecular etiology of chronic pain and offer potential as novel therapeutic targets.

## Figures and Tables

**Table 1:** Role of miRs in pain receptors [98].

Diseases	miRs	Tissues	Targets	Ref
IBS	miR-199 ↓	Human colon; Rat colon/DRG	TRPV1 ↑	[99]
	miR-24 ↑	Human/mouse intestinal mucosa	SERT ↓	[100]
	miR-17-5p ↑	Lumbar spinal cord	STAT3 ↓; gp130 ↑	[101]
	miR-150 ↑ miR-342-3p ↑	Human whole blood	-	[102]
	miR-29a ↑	Human small bowel and colon; human blood macrovesicles	Glutamate ammonia ligase ↓	[103]
	miR-144 ↑	Rat distal colonic epithelial cells	Occludin ↓; ZO1 ↓	[104]
Endometriosis	miR-9 ↓; miR-34 ↓	Human endometrial tissues	-	[105]
	miR-142-3p ↑	Endometrial stroma cells	Steroid sulfatase ↓; gp130 ↓	[106]
	miR-29 ↑; miR-181 ↑; let-7 ↑	ox-LDL-treated human endometrial cell lines	NGF ↑; IL-6 ↑; PTGES3 ↑	[107]
	miR-122 ↑; miR-199a ↑	Serum; peritoneal fluid	-	[108]
BPS/IC	miR-449b ↑ miR-500 ↑	Bladder smooth muscle cells	NK1 receptor↓	[109]
	miR-199a-5p ↑	Bladder smooth muscle; Mature bladder urothelium; Primary urothelial culture	LIN7C ↓; ARHGAP12 ↓; PALS1 ↓; RND1↓; PVRL1 ↓	[110]
	miR-214 ↓	Postmenopausal women’s bladder tissue; Ovariectomized rats’ APMSCs	Mfn2 ↑	[111]
	miR-139-5p ↓	Postmenopausal women’s bladder tissue	LPAR4 ↑	[112]
	miR-181a ↑	Rat spinal cord	GABA_A_ ↓	[113]
	miR-92b-3p ↑	Rat spinal cord	KCC2 ↓; VGAT ↓	[114]

**Table 2:** miRNAs and gene association.

miRs	Targets	Role	Ref
miR-1	Sodium voltage-gated channel alpha subunit 1 (SCN1A)	Involved in controlling neuronal excitability, and neuropathic pain has been linked to its dysfunction	[49]
miR-21	Programmed cell death 4 (PDCD4), Sprouty homolog 2 (SPRY2), and others	Implicated in neuroinflammation and it has been found to be upregulated in models of neuropathic pain	[161]
miR-23b	Prostaglandin-endoperoxide synthase 2 (PTGS2/COX-2)	Involved in controlling inflammatory pathways; a malfunction in this regard could be the cause of inflammatory pain	[162]
miR-124	Signal transducer and activator of transcription 3 (STAT3)	Linked to neuroinflammation and microglial activation; dysregulation may be a factor in neuropathic pain	[163]
miR-155	Suppressor of cytokine signaling 1 (SOCS1), SH2-containing inositol phosphatase 1 (SHIP1), and others	Involved in immune response and neuroinflammatory modulation; elevated in chronic pain models	[164]
miR-146a	Interleukin-1 receptor-associated kinase 1 (IRAK1) and tumor necrosis factor receptor-associated factor 6 (TRAF6)	Involved in the regulation of immune responses and inflammation; dysregulation may contribute to chronic pain conditions	[165]
miR-29	Collagens, involved in extracellular matrix regulation	Linked to the control of extracellular matrix components and fibromyalgia; dysregulation may be a factor in the abnormalities of connective tissue in chronic pain	[124]
miR-30a	Serine/threonine-protein kinase WNK1	Connected to WNK1 expression variation, which may affect sensitivity to pain	[166]
miR-128	Voltage-gated sodium channel alpha subunit 2 (SCN2A)	Involved in controlling the excitability of neurons; dysregulation could lead to neuropathic pain	[167]
